# Microvillous inclusion disease (microvillous atrophy)

**DOI:** 10.1186/1750-1172-1-22

**Published:** 2006-06-26

**Authors:** Frank M Ruemmele, Jacques Schmitz, Olivier Goulet

**Affiliations:** 1 INSERM EMI 0212, Pediatric Gastroenterology, Hepatology and Nutrition, Hôpital Necker-Enfants Malades, 149 Rue de Sèvres, 75743 Paris Cedex 15, France

## Abstract

Microvillous inclusion disease (MVID) or microvillous atrophy is a congenital disorder of the intestinal epithelial cells that presents with persistent life-threatening watery diarrhea and is characterized by morphological enterocyte abnormalities. MVID manifests either in the first days of life (early-onset form) or in the first two months (late-onset form) of life. MVID is a very rare disorder of unknown origin, probably transmitted as an autosomal recessive trait. To date, no prevalence data are available. Ultrastructural analyses reveal: 1) a partial to total atrophy of microvilli on mature enterocytes with apical accumulation of numerous secretory granules in immature enterocytes; 2) the highly characteristic inclusion bodies containing rudimentary or fully differentiated microvilli in mature enterocytes. Light microscopy shows accumulation of PAS-positive granules at the apical pole of immature enterocytes, together with atrophic band indicating microvillus atrophy and, in parallel, an intracellular PAS or CD10 positive line (marking the microvillous inclusion bodies seen on electron microscopy). Intestinal failure secondary to diarrhea is definitive. To date, no curative therapy exists and children with MVID are totally dependent on parenteral nutrition. Long-term outcome is generally poor, due to metabolic decompensation, repeated states of dehydration, infectious and liver complications related to the parenteral nutrition. As MVID is a very rare disorder, which is extremely difficult to diagnose and manage, children with MVID should be transferred to specialized pediatric gastro-intestinal centers, if possible, a center equipped to perform small bowel transplantation. Early small bowel transplantation resulting in intestinal autonomy gives new hope for disease management and outcome.

## Disease name and synonyms

Microvillous inclusion disease

Microvillous atrophy

Congenital enteropathy

Congenital familial protracted diarrhea with enterocyte brush-border abnormalities

## Definition and diagnostic criteria

Microvillous inclusion disease (MVID) or microvillous atrophy (MVA) is a congenital and constitutive disorder of intestinal epithelial cells [1-6]. It is characterized by the neonatal onset of abundant watery diarrhea persisting despite total bowel rest. Onset most often occurs within the first days of life. Microvillous atrophy, first described in 1978 [7], was the first clinical entity identified on a morphological basis as being responsible for the so-called protracted or intractable diarrhea syndrome. The diagnosis is based on typical morphological abnormalities detected through a combination of light and electron microscopic (EM) analyses of small bowel biopsies [8-14]. Standard histology reveals a variable degree of villous atrophy without marked crypt hyperplasia, in addition to abnormal periodic-acid schiff (PAS) positive secretory granules accumulating in the apical cytoplasm of mature enterocytes and an altered (atrophic after PAS staining) enterocyte brush border membrane. These findings are completed by EM with the detection of atrophic or completely absent microvilli on mature enterocytes (see below) along with so-called microvillous inclusions (vacuoles lined by microvilli) and the finding of large PAS positive granules in immature enterocytes.

### Clinical presentation

Pregnancy and delivery are uneventful; in general, there is no notion of polyhydramnios except in rare isolated cases. Severe watery diarrhea starts within the first days of life [1-4,6,7]. This diarrhea becomes so abundant, that within 24 h the children can loose up to 30% of their body weight, resulting in profound metabolic acidosis and severe dehydration. MVID is most often severe and life-threatening. Accurate quantification of the stool volumes reveals 150 to over 300 ml/kg/d, with a high sodium content (approximately 100 mmol/L). Complete and prolonged bowel rest allows to reduce stool volume moderately, but volumes nearly always remain above 150 ml/kg/day [4]. Inappropriate parenteral nutrition with steadily increasing intravenous fluids may significantly aggravate stool output. No additional clinical signs are associated with MVID; in particular, there are no malformations or involvement of other organs such as liver, kidney etc. However, a small number of children has a massive pruritus secondary to marked elevations in the concentrations of biliary acids in the blood. Initially, no specific findings can be detected except enormous abdominal distension with fluid-filled intestinal and colonic loops. All children with congenital MVID urgently require total parental nutrition (TPN), which often causes rapidly evolving cholestasis and liver disease. A detailed multicenter analysis of 23 patients with MVID [4] allowed two different forms and presentations of MVID to be distinguished on a clinical and morphological basis: congenital early-onset MVID (starting within the first days of life), and late-onset MVID (with first symptoms appearing after two or three months of life).

### Biological testing

Secondary to the marked diarrhea, children with MVID rapidly develop metabolic acidosis and signs of hypotonic dehydration. No other biological signs are associated with this disorder, however, most children are at risk of developing cholestasis and liver failure. Stool examination reveals fecal sodium concentrations between 100 and 130 mmol/L, normal alpha-1 antitrypsin clearance, and no fecal inflammatory parameters.

### Endoscopy/Biopsies

**Figure 1:**
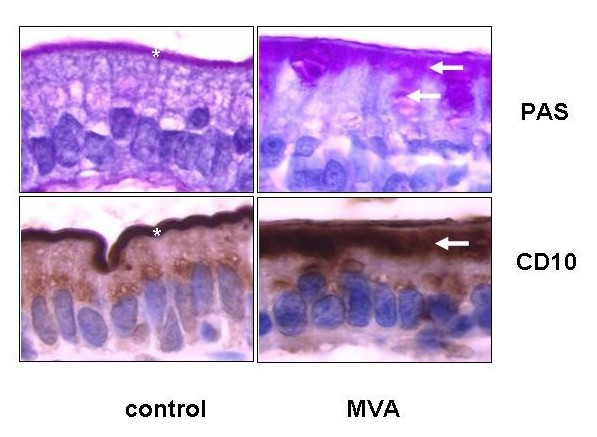
High power magnification of a duodenal section of a patient with typical microvillous inclusion disease or microvillous atrophy (MVA) after periodic schiff acid (PAS) staining or anti-CD10 immunohistochemistry. As shown on both panels compared to normal controls, in MVA an enlarged intracytoplasmic band (arrow) along the apical pole of enterocytes is observed along with an atrophic band instead of the normally well-defined small line representing the brush border (asterix).

The gold standard in the diagnosis of MVID is a combined light/electron microscopic histological analysis (Figure 1) of small bowel biopsies obtained during diagnostic gastrointestinal endoscopy. Macroscopic endoscopic analysis of the entire gastro-intestinal tract remains completely normal, besides non-specific minimal alterations, such as mild mucosal erythema or, in rare cases, indirect signs of villous atrophy. In contrast, histological analysis reveals major alterations of the entire small bowel and, to a lesser degree, also of the colon. Standard histology shows a variable degree of villous atrophy without marked crypt hyperplasia, appearing as "thin mucosa" [11,15]. The accumulation of PAS positive granules within the apical cytoplasm of immature enterocytes in the upper crypt is highly characteristic of MVID [13,14]. In parallel, on PAS staining (light microscopy), the brush border membrane looks pathological, with an enlarged intracytoplasmic band along the apical pole of enterocytes (corresponding to autophagocytic vacuoles and microvillous inclusion bodies revealed by EM) and an atrophic band instead of the normally well-defined small line representing the brush border (Figure 1).

New immuno-staining techniques directed against CD10, a neutral membrane-associated peptidase, can further help the diagnosis of MVID [9,14], since the small linear band of the brush border appears markedly enlarged and as a double band in MVID patients. This abnormal staining pattern (PAS or CD10) on the apical pole of enterocytes appears first in upper crypt epithelial cells in congenital MVID with early onset, whereas late-onset MVID has abnormal enterocyte structures within the lower villous part. Based on the morphological presentation of abnormal PAS stain in low crypt cells, an atypical form of MVID was also described. On EM, mature enterocytes show a reduced to completely absent microvillus profile on the apical membrane, and increased numbers of autophagic granules [8]. In addition, the diagnostic and characteristic microvillus inclusion bodies are easily distinguishable by EM. In contrast to mature enterocytes, crypt cells appear almost normal on EM apart from increased number of secretory granules. Thus, the diagnosis of MVID is difficult and should be performed or at least confirmed by particularly skilled pathologists who most often can make this diagnosis on light microscopy after PAS or CD10 staining (characteristic alterations in the apical pole of immature and mature enterocytes). However, the diagnosis is formally confirmed by the finding of microvillous inclusion bodies within the cytoplasm along with atrophic microvilli on EM. Since microvilli on immature crypt cells are most often normal, isolated EM analysis of these cells should not be performed as it could lead to a false negative diagnosis. In addition, the isolated finding of rudimentary or absent microvilli on enterocytes is also not sufficient to diagnose MVID with certitude, as discussed.

## Complications

As a result of the severe diarrhea, acute episodes of dehydration and metabolic decompensation are common complications of MVID. Hypovolemia often causes temporary ischemia, therefore, neurological and psychological symptoms such as developmental retardation can occur [4]. Impaired renal function is also a frequent complication, together with nephrocalcinosis. TPN is extremely difficult to adapt and requires vast experience, especially for the initial stabilization of small babies with MVID. Major complications of TPN such as cholestasis or liver failure are normally avoidable, but still very frequent. Infectious complications of the central catheter resulting in sepsis are the most frequent cause of death, followed by liver failure.

## Outcome

MVID is a constitutive intestinal epithelial cell disease that causes an irreversible diarrheal disorder leading to permanent and definitive intestinal failure [4,7,16]. Children with MVID are dependent on exclusive parenteral nutrition throughout their lives. Oral alimentation and appropriate oral caloric intake are impossible. There is no hope for improvement with age. A large number of patients do not survive the first three years of life as a result of infectious complications or rapid evolution of liver failure. Those children with MVID who survive often have mental and statural retardation, and renal complications. In contrast to children with early-onset MVID, the diarrhea is often less severe in children with the late-onset form of the disease. With age, children with late-onset MVID can acquire partial intestinal autonomy, resulting in a reduction of the number of perfusions of parental nutrition to one or two a week.

## Etiology

The precise etiology of MVID is still unknown. A major defect in membrane trafficking in enterocytes has been proposed as pathogenetic mechanism of MVID, probably secondary to an altered structure of the cytoskeleton [17]. However, the observation of morphologically normal microvilli on immature crypt cells in children with MVID indicate that the microvillous changes seen in differentiating and mature cells are of a secondary nature or are a consequence of yet unidentified events within the cell. These events could include membrane recycling or mechanisms controlling endo- or exocytosis [18,19]. However, analysis of the membrane targeting of disaccharidases, such as sucrase-isomaltase, revealed no abnormalities of the direct or indirect constitutive pathway. Very recent observations indicate a selective defect in glycoprotein exocytosis in patients with MVID [20]. These findings need further confirmation. Another hypothesis suggesting a defect in the autophagocytosis pathway was recently proposed to explain the morphological and functional abnormalities in MVID [21].

## Differential diagnosis

Epithelial dysplasia (tufting enteropathy), inflammatory bowel disorder, autoimmune enteropathies, chloride or sodium diarrhea, Na-H-exchange deficiency, glucose-galactose malabsorption, sucrase-isomaltase deficiency, rarely intestinal pseudo-obstruction syndrome or motility disorders.

## Epidemiology

MVID is an extremely rare congenital disorder. To date, no prevalence data are available, however, it can be estimated that there are no more than a few hundred children with MVID in Europe. The prevalence is higher in countries with a high degree of consanguinity, suggesting autosomal recessive transmission.

## Genetic counseling

The observations that the incidence of MVID is higher in families with a pre-existing case of MVID and that there is a high rate of consanguinity in parents of children with MVID, clearly indicate a genetic basis for this disorder, which is probably inherited on an autosomal recessive basis. It is particularly striking to note the large number of children of Turkish origin with MVID and a high degree of consanguinity in this population. As genetic defect has not been identified, no genetic counseling or prenatal diagnosis is possible.

## Management including treatment

To date, no causal treatment exists for MVID. Trials with anti-inflammatory drugs including steroids and anti-secretory medications (such as sandostatin or loperamide), did not significantly change stool volumes over a prolonged period [4]. Therefore, all patients are dependent on supportive measures such as parenteral nutrition, which is the only way of stabilizing them. However, this treatment is often difficult to administer successfully as the diarrhea is very abundant and the patients can rapidly succumb to metabolic decompensation. Therefore, it is important that children with MVID are transferred to highly specialized pediatric gastroenterology centers. As discussed, the long-term outcome is rather poor for children treated with parenteral nutrition. New treatment strategies for the management of MVID are needed. Intestinal transplantation is a clear alternative to parenteral nutrition for children with MVID [16,22-24]. It can be performed as isolated small bowel or combined liver-small bowel transplantation, if significant liver disease exists. A recent study revealed that the outcome for children who had undergone small bowel transplantation for MVID was much better than that for children undergoing small bowel transplantation for other indications [16]. Based on this observation and the fact that prolonged parenteral nutrition has a rather poor outcome, it is now appropriate to consider early small bowel transplantation as a first choice treatment of early-onset MVID, allowing the patients to obtain full intestinal autonomy. However, it is hoped that a curative treatment will be available in the near future.
